# Reflections on the Brain Conference 2023

**DOI:** 10.1093/braincomms/fcad102

**Published:** 2023-05-03

**Authors:** Elizabeth Simzer

**Affiliations:** Edinburgh, UK

## Abstract

Our acting Scientific Editor, Elizabeth Simzer, highlights a session from the third annual Brain Conference held on 16 March 2023.

**Graphical Abstract:**
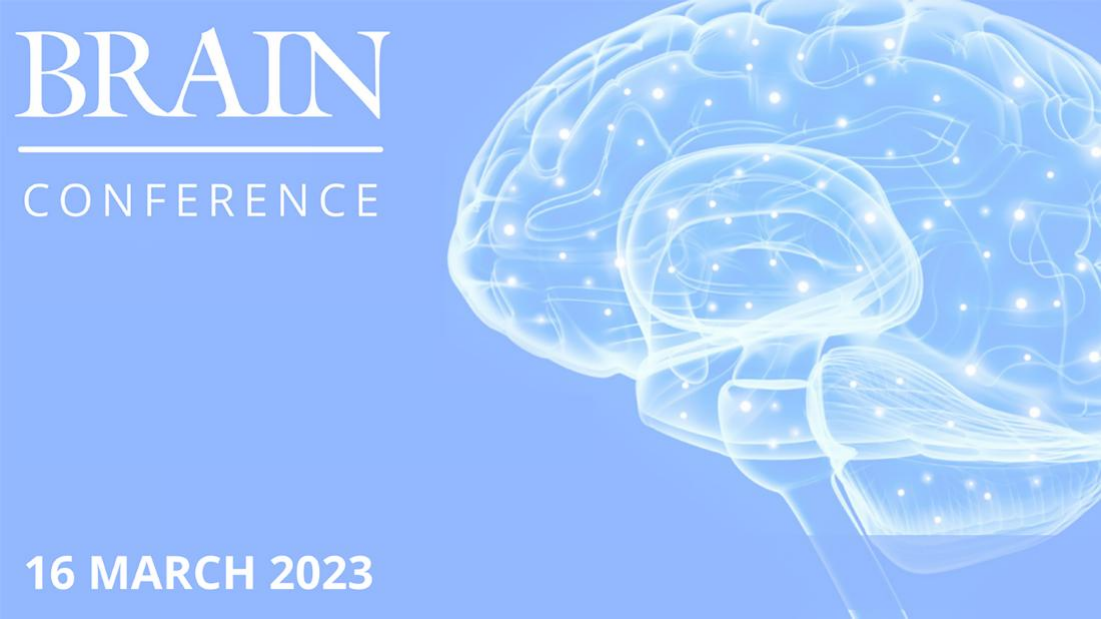


Welcome to Volume 5, Issue 3 of *Brain Communications*. In this editorial, I want to highlight a session from the third annual Brain Conference held on 16 March 2023. But first, we would like to congratulate our early career researcher paper prize^1^ winner George Thomas, who gave a fascinating presentation on his winning paper ‘Top-down and bottom-up connectivity changes in Parkinson’s disease visual hallucinators’.^2^ George stood out from the many competitive papers for his cutting-edge, robust research and contribution to promoting open access science through shared code and group-level data. Congratulations George! Keep an eye out for excellent papers by deserving early career researchers in *Brain Communications* this year, as we will be rerunning the prize with a call for nominations in January 2024.

The Brain Conference was started in 2021 by the Guarantors of Brain to actively promote ‘teaching, education, and research in neurology and related disciplines’,^3^ and has been successful in this mission over the past 3 years.

This year’s conference covered a vast range of neuroscience disciplines, featuring scientists and clinicians from around the globe who curated and chaired a range of insightful sessions. Specifically, the ‘Central nervous system diseases in low-and middle-income countries (LMIC)’ session chaired by Professor Alfred Njamnshi, one of *Brain Communications’* Associate Editors, and Professor Gagandeep Singh highlighted the burden and challenges of epilepsy and other neurological diseases faced by LMIC.

Affecting over 50 million people worldwide, epilepsy is a central nervous system disorder characterized by the occurrence of unprovoked seizures. Despite its global prevalence, over 80% of people living with epilepsy live in LMIC.^4^ The World Health Organization has reported that 70% of people with epilepsy can be seizure-free with treatment; however, three-quarters of people living with epilepsy in LMIC do not have access to treatment, increasing their risk of premature mortality by three times.^4^ As highlighted by Professor Gagandeep Singh, we must focus on ameliorating this treatment gap to ‘prevent several premature deaths [and] reduce disability, injuries, and some of the psychosocial consequences of epilepsy’ (G. Singh, Brain Conference; 16 March 2023).

But how do we address and begin to amend this immense treatment gap?

A variety of challenges and viable solutions were explored throughout the session. Professor Paul Seke touched on the challenges of research in LMIC, such as insufficient financial resources and skilled personnel, pronounced gender disparities, and language barriers, which limit epilepsy research to experimental models that are relatively inexpensive and easy to implement. Professor Gagandeep Singh focused on the necessity of primary care for epilepsy and the ways in which epilepsy treatment needs to be improvised and adapted on a community-by-community basis. Professor Ley Sander emphasized why primary prevention is a viable approach to reduce the resulting epilepsy burden, highlighting the social determinants of health perpetuating these issues.

Overall, it is a nuanced and complex issue that cannot be solved by a one-size-fits-all approach. Instead, as stated by Professor Singh, it is essential that we ‘think globally [and] act locally to improvise global solutions and adapt them to the local context and setting’ (G. Singh, Brain Conference; 16 March 2023). Although there is still much work to do, it is important that organizations like Guarantors of Brain continue to create a space for this dialogue and bring attention to these issues. However, it is up to us as clinicians, scientists and fellow human beings to continue educating ourselves on these topics, to better support and advocate for change.

If you would like to view these insightful speakers or any of the other sessions that took place at the Brain Conference 2023, there is still time! All the excellent sessions are available to view on demand until 30 June 2023. Registration is available here: https://brain-conference-2023.idloom.events/the-brain-conference-2023

The cover of this issue comes from our Editor in Chief, Professor Tara Spires-Jones, and is a photo of electron microscopy nickel grids floating on droplets of liquid during an immuno-staining protocol. You will be thrilled to know that the immuno-gold labelling of tau worked in this experiment.
